# Psychological Benefits of Purchasing Home Meal Replacement in the Context of Eco-Friendly TV Home Shopping Broadcast: The Moderating Role of Personal Norm

**DOI:** 10.3390/ijerph19137759

**Published:** 2022-06-24

**Authors:** Heather Markham Kim, In-Hye Lee, Kyuhyeon Joo, JungHoon (Jay) Lee, Jinsoo Hwang

**Affiliations:** 1 The College of Hospitality and Tourism Management, Sejong University, Seoul 143747, Korea; heather.markham923@gmail.com (H.M.K.); khjoo@sju.ac.kr (K.J.); 2 School of Theatre & Film Art, Kyungsung University, Busan 48434, Korea; inhye@ks.ac.kr; 3 School of Hospitality Leadership, East Carolina University, 306 Rivers Building, Greenville, NC 27858-4353, USA; leejun@ecu.edu

**Keywords:** eco-friendly, TV home shopping broadcast, psychological benefits, personal norm, theory of planned behavior, home meal replacement

## Abstract

This study observed the relationship between psychological benefits and the theory of planned behavior (TPB) in the context of an eco-friendly TV home shopping broadcasts. The theoretical framework was enhanced even further by examining the moderating role of personal norm on proenvironmental attitudes in the TV home shopping context. An online survey was conducted with Korean customers who had purchased home meal replacement (HMR) products from a TV home shopping broadcast within the past 6 months. A total of 305 samples were collected and used for the data analysis. All six of the hypotheses in the psychological benefits and TPB model were supported, meaning all constructs of psychological benefits, including warm glow, self-expressive benefits, and nature experiences, impacted TPB and behavioral intentions. Furthermore, personal norm had a moderating role in the relationship between warm glow and attitude. This research provides significant theoretical and managerial implications for the home shopping industry.

## 1. Introduction

A combination of the occurrence of the COVID-19 pandemic and an increase in peoples’ awareness regarding environmental issues has made a significant impact on consumer trends. Physical distancing measures were set in place to help stop the spread of the infection, and with more people staying at home, reductions in pollution emissions were observed [1]. Consumers were able to witness our society’s impact on the environment, and this naturally led to an increase in awareness of eco-friendly products and pro-environmental behaviors. For example, many people were forced to stay at home due to social distancing measures, and even daily shopping needs were purchased through contactless services. Social distancing measures led to an escalation in ecommerce shopping, including TV home shopping broadcasting [2]. The pandemic also had a significantly negative impact on the global economy and international trading [3]. Therefore, the need for sustainable economic development was brought to light, and small and medium enterprises (SMEs) are recognized as a necessity for improved international trade in both developed and developing countries [4]. Home shopping networks are major vessels for SMEs to sell their products. For example, a South Korean government-run home shopping channel called Gongyong Home Shopping sells Korean SME products [5]. In the US, well-known everyday brands have strong relationships with the most popular home shopping broadcasting companies, including QVC and HSN, making it appealing for consumers to shop from TV home shopping [6]. A similar trend has occurred in South Korea as well. According to the 2020 Consumer Behavior Survey for Food, the proportion of purchasing processed food through online malls and TV home shopping increased from 4% in 2019 to 11.4% last year, and 55.6% of respondents referred to themselves as eco-friendly food buyers [7]. Particularly, the general food category performance from TV home shopping broadcasts in Korea was profitable last year as the trend of home-cooked meal consumption and the demand for contactless services coincided. Trends in Korea that have influenced the TV home shopping broadcasting industry are the increase in single-person households and dual-income families, which has resulted in steady sales of home meal replacement (HMR) products through TV home shopping broadcasts [4,8]. Sales in the Korean HMR market are forecasted to advance to five trillion KRW by 2022, and companies are investing in research and development of HMR products [9]. The HMR trend is not just limited to Korea, as food manufacturers and grocery stores across the globe are in the midst of creating HMR products for market expansion [10].

Not only have industries seen a change in contactless services, but also the increase in environmental protection interest has made an impact. According to the Stifel and Morning Consult survey [11], 71% of US adults between the ages of 18 and 55 focused more on product sustainability compared with the previous year [11]. Eco-friendly TV home shopping broadcasts will appeal even more to consumers who seek sustainable products, whether they adopt eco-friendly policies from the TV broadcasting company directly or whether they offer eco-friendly products on their programs. For example, one of the most popular TV home shopping broadcast brands in the US, QVC, has recently launched a completely sustainable fashion brand called Seed to Style, which can only be purchased through QVC [12].

More importantly, using green brands, including eco-friendly TV home shopping broadcasts, helps consumers reap psychological benefits [13,14]. Psychological benefits refer to having trust or confidence in another individual or group that reduces anxiety and increases freedom from worry [15]. Customers who perceive psychological benefits while using green brands may feel a sense of comfort. Therefore, when consumers use eco-friendly TV home shopping broadcasts, they will most likely perceive a high degree of psychological benefits. Despite the significance of the psychological benefits of eco-friendly services, there is no current research that has studied the psychological benefits in the context of an eco-friendly TV home shopping broadcast, and this study attempted to fill that research gap.

In addition, in order to predict consumers’ eco-friendly behavioral intentions, this study applied the theory of planned behavior (TPB) to an eco-friendly TV home shopping broadcast. This model is adequate in clarifying the reasons an individual engages in eco-friendly behaviors based on volitional and nonvolitional factors and helps predict motivation triggers as well [16]. Even though TPB is an important factor in determining behavioral intentions in green brands, no studies have attempted to research the effects of TPB in the context of an eco-friendly TV home shopping broadcast.

Lastly, the moderating role of personal norm was also explored in this study. Personal norm has been found to have a positive relationship with behavioral intentions in eco-friendly settings [17]. Prior studies have found that personal norms help to predict attitudes [18] and, in particular, attitudes related to the environment [19]. When stimulated, personal norms have a direct impact on eco-friendly behaviors [20]. It was revealed that personal norms play a moderating role in compliance with proenvironmental social norms, and the activation of personal norms can fill the void of social norm receptiveness [21]. The moderating role of personal norm has also been found in the context of environmentally framed eWOM messages [22]. In this study, negative reviews of a high environmental impact product vs. negative reviews of a low environmental impact product were perceived to be more useful by consumers with high personal norms, and consumers with low personal norms did not show significant responses [22]. The negative reviews perceived by high personal norm consumers, in turn, negatively impacted attitude and purchase intention. Thus, it can be assumed that a higher level of personal norm would increase the probability that consumers would participate in proenvironmental behaviors [23]. Although personal norm plays a significant role in eco-friendly contexts, no study has investigated the moderating role of personal norm in the context of an eco-friendly TV home shopping broadcast.

Therefore, the objectives of this study were to investigate (1) the impact of psychological benefits on attitude, (2) the influence of attitude, subjective norm, and perceived behavioral control in forecasting behavioral intentions, and (3) the moderating role of personal norm in the relationship between psychological benefits and attitude in the context of an eco-friendly TV home shopping broadcast.

## 2. Literature Review

The concepts of the proposed model for this study are discussed in detail in the following literature review. After discussing each concept, the reasoning behind the hypotheses development is also explained.

### 2.1. Eco-Friendly TV Home Shopping Broadcast

Television home shopping broadcasts are television programs that feature products sold by on-air hosts, and viewers are able to place orders through their telephones, television remotes, or smartphone applications. Home shopping broadcasting offers a more interactive experience for consumers compared with online shopping [24]. Sales through home shopping broadcasting channels have recently increased due to the demand for contactless shopping after the emergence of the COVID-19 pandemic. In the United States, the pandemic caused several offline retailers, including J.C. Penny, Sears, and Neiman Marcus, to close down as the need for online retail channels increased [25]. In South Korea, the home shopping application Home Shopping Moa reported that food purchases such as home meal replacement (HMR) products increased by more than 40% when the government increased the social distancing measure to level four [26]. The rise in sales of HMR can be explained by the fact that consumers were cooking more at home due to the pandemic. Along with HMR products, sales for seafood products rose 136%, home convenience food sales rose 63.1%, and health food sales rose 44.5% [27]. Not only have consumers across the world focused on the impacts of the pandemic but also interest in protecting the environment has risen significantly in recent years.

According to Chen and Tung [28], consumers have become more conscious about making environmentally friendly choices and, in turn, search for eco-friendly products, and are even willing to pay more for these products. When companies adopt eco-friendly practices, they not only contribute to helping the environment but also may develop a competitive advantage, improve brand image, expand new markets, and improve the overall value of products [29]. The commitment to the environment and becoming greener can also be seen in the broadcasting industry. For example, the British Broadcasting Corporation (BBC) has reduced single-use plastic utensils by more than half a million, actively strives to install zero-emission generators, uses LED lighting to save energy, and uses heated seats instead of fan heaters in the winter [30]. These eco-friendly broadcasting practices can also be found in home shopping broadcasting.

The NS Home Shopping broadcasting company is based out of South Korea, and according to its website, the company has been committed to creating an environmentally friendly broadcasting network since March 2020. Just like the BBC, NS Home Shopping has replaced the halogen lights in their four studios with LED lights, which can be used 100 times longer than halogen lights [31]. Being able to use LED lights for a longer period of time helps to reduce the amount of waste produced over time. Another technique NS Home Shopping has established is a zero-gas consumption policy. Moreover, the company has switched to using paper ice packs for refrigerated or frozen delivery products [32]. An additional home shopping broadcasting company in South Korea that has committed to adopting green practices is GS Shop. According to their website, GS Shop started using 100% recyclable paper for packaging in place of the nonwoven and polyvinyl fabric previously used. Their goal is to reduce polyvinyl waste by 3 tons and nonwoven waste by 1.4 tons [33]. Another method GS Shop is using is to use water ice packs for frozen food and paper tape or no tape in the packaging of delivery products. Tags for products can create unnecessary waste, and in order to combat this waste, GS Shop has started using eco-friendly tags that are made of recycled paper for certain products. As a result of implementing these green practices, products with eco-friendly packaging materials surpassed 30% in the first quarter of this year [34].

### 2.2. Psychological Benefits of Using an Eco-Friendly TV Home Shopping Broadcast

The psychological benefits of using a product or brand can be described when consumers develop a sense of trust, comfort, and reduced stress levels when using a particular product [35]. This same idea can be applied to eco-friendly services and products. Consumers feel that they are helping to save the environment when they use eco-friendly brands and, therefore, feel a sense of comfort [13]. This concept has been confirmed in other industries as well. For example, Hartmann and Apaolaza-Ibanez [13] reported that when consumers experience the psychological benefits of using eco-friendly brands, they tend to have a more positive attitude toward the brand. Han et al. [36] found consumers developed green trust when they were satisfied with the benefits of using eco-friendly museums. According to Hwang et al. [14], consumers felt comfort when using drone food delivery services if they acknowledged that drone services helped the environment. Previous studies have determined psychological benefits consist of three subcategories, including warm glow, self-expressive benefits, and nature experiences (e.g., [13]).

First, warm glow is defined as “satisfaction that goes beyond the benefits derived from aggregate provision of a public good through pro-environmental behavior” [37] (p. 239). In reference to eco-friendly products and services, consumers experience a feeling of warm glow when using these products and services because they feel good about contributing to protecting the environment [13]. That is, they experience moral satisfaction intrinsically and are willing to pay a premium price for green products in order to feel good about themselves [38]. Brand placement is an unobtrusive form of television advertising, and previous research found green brand placement positively affected brand attitude and purchase intentions because green brands are perceived as having warmth due to their proenvironmental characteristics [39]. Modern green advertising, including TV advertisements, is an effective tool to influence purchase intention among altruistic consumers [21]. Therefore, it can be assumed that consumers of eco-friendly TV home shopping broadcasts will experience warm glow because of the proenvironmental traits of the broadcast. The height to which people experience warm glow is dependent on their level of social responsibility [40]. The higher the level of social responsibility, the more people will feel warm glow, and when consumers have a lower level of social responsibility, they will feel less warm glow [37,41].

Prior studies have found warm glow has a direct impact on consumers’ attitudes toward a brand or product. For example, Hartmann and Apaolaza-Ibanez [13] found that consumers who felt a warm glow when exposed to experimental green energy brands had a positive influence on their attitude toward the brand. According to Boobalan, Nachimuthu, and Sivakumaran [42], when consumers purchase organic foods, they feel a warm glow, which positively affects attitude. In addition, Bhutto et al. [43] discovered that consumers who purchased energy-efficient home appliances felt warm glow benefits, resulting in positive effects on attitude toward the products. Therefore, if consumers feel a sense of warm glow when purchasing products from an eco-friendly TV home shopping broadcast, they may develop a positive attitude. Thus, the first hypothesis was proposed.

**Hypothesis** **1** **(H1).** *Warm glow has a positive effect on attitude.*

The second subcategory of psychological benefits are self-expressive benefits. Self-expressive benefits have been derived from two theories, including signaling theory and self-congruity theory. Signaling theory refers to the practice of participating in behaviors that show specific characteristics or preferences to others in order to express information about oneself [13]. In the case of environmentally friendly services, consumers feel high levels of self-expressive benefits when they express their desires to protect the environment by purchasing eco-friendly products or services [41]. The second theory, the self-congruity theory, states consumers tend to purchase products that reflect their self-image or even the image they strive to obtain [44]. Consumers with positive green images are more likely to purchase eco-friendly products and also reflect a positive image to society [45]. Prior studies have found similar results. For instance, Hwang et al. [14] found that self-expressive benefits significantly impacted positive and negative emotions in the context of drone food delivery services. They discovered that when consumers were able to express their interests in environmental protection while using drone food delivery services, they had a good feeling about drone food delivery services overall. Similar to these results, Hwang and Kim [46] discovered that self-expressive benefits had a positive relationship with attitude. In their study, they discovered that consumers who were able to express their interest in environmental issues by visiting edible insect restaurants had positive attitudes toward the restaurant brand. From a theoretical background, it can be inferred that if consumers are able to feel self-expressive benefits when purchasing products from an eco-friendly TV home broadcast, the consumer’s attitude toward the brand should be enhanced. Therefore, the following hypothesis was proposed.

**Hypothesis** **2** **(H2).** *Self-expressive benefits have a positive effect on attitude.*

The third component of psychological benefits is nature experiences. The concept of nature experiences is particularly significant for eco-friendly services, as nature directly relates to the environment. It is human nature for people to want to spend time in natural settings in order to improve their overall emotional and physical well-being and reduce stress levels [41]. People who have many nature experiences are more likely to have tendencies to want to protect the environment. Therefore, many brands, especially green brands, use beautiful natural imagery in their advertisements to encourage buyers to purchase eco-friendly products and services [14]. Hartmann and Apaolaza-Ibanez [13] found that consumers who viewed advertisements related to nature and felt a certain degree of nature experiences had a significant impact on brand attitude. Hwang and Choi [41] concluded that when customers of an environmentally friendly airline company felt a sense of closeness to nature, they were more likely to develop a positive image of the airline company. Consumers with a high sense of nature experiences will have more tendencies to want to protect the environment and, in turn, will have positive attitudes toward eco-friendly TV home shopping broadcasts. Hence, the following hypothesis was proposed.

**Hypothesis** **3** **(H3).** *Nature experiences have a positive effect on attitude.*

### 2.3. Theory of Planned Behavior

The theory of planned behavior (TPB) is an expansion of the theory of reasoned action (TRA) and is one of the most widely accepted models in researching behavioral intentions [47]. In anticipating consumer behavior, the TPB is a more appropriate model because the TRA only considers volitional factors, whereas the TPB also includes behavior determined by nonvolitional factors, such as resources [48]. Although the TPB was proposed more than three decades ago, numerous researchers have been proving this framework in consumer behavior until recently (e.g., [42,49,50]). For instance, Boobalan et al. [42] applied the TPB model successfully to investigate the motivations for consumers to purchase organic foods. Particularly, the TPB is an appropriate model to use in eco-friendly research. Wu et al. [51] discovered that the TPB model helped to understand how consumers come to the decision to purchase green residences in China. Moreover, in the context of green restaurants, Moon [49] used the TPB model successfully to research the reasoning behind consumers choosing green restaurants in Korea. In the context of this study, according to the TPB, even if a customer is satisfied and supports the products and policies of an eco-friendly TV home shopping broadcast, if the price of the products is not affordable, the consumer will not be able to use the products. The TPB model helps to explain behaviors with both volitional and nonvolitional factors, therefore making it more sufficient for this study.

The TPB model includes the following concepts: attitude, subjective norm, perceived behavioral control, and behavioral intentions. First, attitude refers to the psychological emotional expression that determines an individual’s positive or negative evaluation of an entity or behavior [28,52]. That is, the most important factor in forming attitude is the consumer’s past experience [53]. Second, subjective norms are defined as the level to which a person feels social pressure to engage or not engage in a particular behavior [47]. That is, the expectations of friends or family members may influence an individual’s behavior. A person with a high level of subjective norm will most likely conform to the expectations of people or groups around them [53]. Third, perceived behavioral control is defined as “people’s perception of the ease or difficulty of performing the behavior of interest” [47] (p. 183). If an individual feels they cannot complete a certain behavior, they are considered to have a low level of perceived behavioral control. Vice versa, an individual who feels they are capable and has the resources to engage in the behavior is said to have a high level of perceived behavioral control [51]. Lastly, behavioral intentions are the willingness to engage in a behavior [47]. It is widely known that behavioral intentions have a direct and important impact on corporate sales [54].

This study proposes Hypotheses 4, 5, and 6 based on the following empirical studies. First, Han et al. [48] also confirmed that the visit intention of consumers of a green hotel was positively influenced by attitude. Hwang and Kim [55] found a positive association between attitude and behavioral intentions in the context of drone food delivery services. Kim and Ryu [56] suggested consumers with a positive attitude had stronger brand attachment and brand loyalty to the robotic coffee shop brand. In the context of an eco-friendly TV home shopping broadcast, if consumers have a positive attitude toward the eco-friendly broadcasting brand, it can be inferred they will more likely purchase products from the home shopping broadcast. Thus, the following hypothesis was proposed.

**Hypothesis** **4** **(H4).** *Attitude has a positive effect on behavioral intentions.*

In addition, Chen and Tung [27] confirmed subjective norm to have a positive relationship with behavioral intentions. Ham et al. [57] suggested subjective norm was a factor in whether consumers purchased green food. Yang et al. [58] found that subjective norms positively influence the intentions of developers’ green procurement behaviors in the construction industry. If an individual has a high level of subjective norm, they will most likely purchase products from an eco-friendly TV home shopping broadcast, as proenvironmental behavior is considered a common good for society. Hence, the following hypothesis was proposed.

**Hypothesis** **5** **(H5).** *Subjective norms have a positive effect on behavioral intentions.*

Lastly, Chen and Tung [28] suggested individuals with more perceived behavioral control of visiting green hotels will have a higher intention to visit green hotels. Han et al. [48] discovered that perceived behavioral control was the important variable of the TPB in determining behavioral intentions in the context of edible insect restaurants. More recently, Choe et al. [53] suggested that perceived behavioral control aids in enhancing behavioral intentions in the context of drone food delivery services. Individuals with strong perceived behavioral control are more likely to purchase products from an eco-friendly TV home shopping broadcast. Thus, the following hypothesis was proposed.

**Hypothesis** **6** **(H6).** *Perceived behavioral control has a positive effect on behavioral intentions.*

### 2.4. Moderating Role of Personal Norm

Personal norms are defined as the “moral obligation to perform or refrain from specific actions” [59] (p. 191). As a result, personal norm is impacted by how an individual identifies themselves with the environment and describes to what level an individual sees themselves as being proenvironment [60]. In understanding the concept of prosocial behaviors, personal norms are considered one of the most significant factors in determining whether an individual engages in prosocial behaviors [61,62]. Individuals with higher personal norms will more likely engage in proenvironmental behaviors, and individuals with lower personal norms will most likely not participate in proenvironmental behaviors. Personal norms are considered the individuals’ ‘inner voice,’ and following one’s personal norms would lead to self-pride, while behaving in a manner that conflicts with one’s personal norms may lead to increased self-depreciation [62].

Prior studies have used the norm-activation model to observe prosocial behavioral intentions and found personal norms positively impact proenvironmental behaviors [17]. Such personal norms are different from perceived social pressure because, in personal norms, the decision to participate in a certain behavior is decided on a personal level [17]. Schultz et al. [59] examined the moderating role of personal norm in the context of water conservation and found people with high personal norms felt a higher obligation to reduce water consumption and evaluated normative information in a different way. Consumers with a higher level of personal norm are more likely to be concerned about the environment and, thus, able to perceive the psychological benefits of purchasing products from an eco-friendly TV home shopping broadcast and have more favorable proenvironmental attitudes. In contrast, consumers with lower levels of personal norms may be less interested in protecting the environment and will not perceive the psychological benefits of using eco-friendly products, and will, in turn, negatively impact attitudes. Therefore, the following hypotheses were proposed.

**Hypothesis** **7a** **(H7a).** *Person norm moderates the relationship between warm glow and attitude.*

**Hypothesis** **7b** **(H7b).** *Personal norm moderates the relationship between self-expressive benefits and attitude.*

**Hypothesis** **7c** **(H7c).** *Personal norm moderates the relationship between nature experiences and attitude.*

### 2.5. Proposed Model

**Figure 1:**
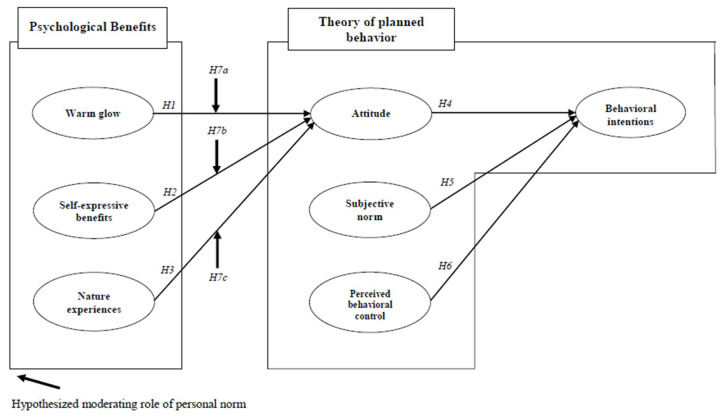
The proposed conceptual model.

Based on these seven hypotheses, the research model is suggested (Figure 1).

## 3. Research Methods

### 3.1. Measurement Items

In order to measure each construct, the measurement items used in this study were adapted from previous studies. Psychological benefits consisted of three subcategories, including warm glow, self-expressive benefits, and nature experiences. All three subcategories of psychological benefits were measured with nine measurement items adopted from Hartmann and Apaolaza-Ibáñez [13] and Hwang et al. [14]. Next, the TPB, which includes attitude, subjective norm, and perceived behavioral control, was measured using nine items from Ajzen [47] and Choe et al. [53]. Finally, behavioral intentions were measured with items adopted from Hwang and Kim [55] and Zeithaml, Berry, and Parasuraman [63]. Based on the construct measurement items, a questionnaire was developed employing a seven-point Likert scale (i.e., 1 = strongly disagree, 7 = strongly agree).

### 3.2. Data Collection

For the purpose of this study, the survey sample included consumers who had purchased HMR products within the past 6 months. The survey was conducted online using email. Prior to beginning the survey, newspaper articles regarding eco-friendly TV home shopping broadcasts were provided to the respondents. The newspaper articles explained how TV home shopping companies were making various efforts to become eco-friendly. The respondents were given enough time to read the newspaper article carefully; participants completed the survey in approximately 10 min. Consequently, a total of 305 surveys were collected, and the data was analyzed using SPSS 23 and AMOS 23 statistical programs.

## 4. Data Analysis

### 4.1. Descriptive Statistics

Table 1 provides the sociodemographic characteristics of the respondents. Among the samples, 151 were female (49.5%) and 154 were male (50.5%). In regard to age, the largest number of respondents were in their fifties (34.1%, *n* = 104), followed by respondents in their forties (28.5%, *n* = 87). As for monthly income, 26.8% of respondents earned less than USD 2000 per month (*n* =82). In terms of marital status, 53.8% of respondents reported being married (*n* = 164), and 44.6% of respondents were single (*n* = 136). Finally, the majority of respondents held a bachelor’s degree (54.8%; *n* = 167).

### 4.2. Measurement Model

Confirmatory factor analysis (CFA) was conducted to test the validity of the measurement constructs of the conceptual model. Based on the results of the analysis, the model fit was found to be acceptable [64]. All of the factor loadings were found to be greater than or equal to 0.769 (Table 2).

All of the average variance extracted (AVE) values presented in Table 3 were found to be greater than the recommended 0.50 limit, meaning the convergent validity of the constructs was supported [65]. The composite reliabilities were greater than 0.70, which indicated internal consistency was validated [66]. Lastly, Table 3 shows that the AVE values were greater than the squared correlations (*R*^2^) between all construct pairs, confirming discriminant validity [67].

### 4.3. Covariance-Based Structural Equation Modeling (CB-SEM)

**Figure 2:**
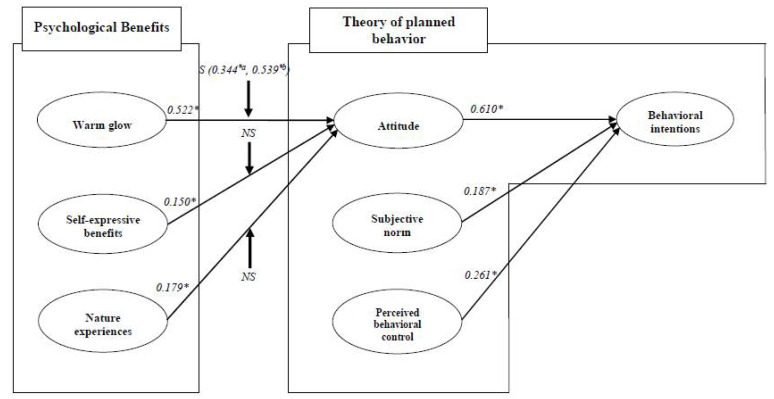
Standardized theoretical path coefficients. Goodness-of-fit statistics: χ^2^ = 427.079, df = 173, χ^2^/df = 2.469, *p* < 0.001, NFI = 0.945, CFI = 0.966, TLI = 0.959, and RMSEA = 0.070. Notes. * *p* < 0.05; S, supported; NS, not supported; *^a^* path coefficient of low personal norm group; *^b^* path coefficient of high personal norm group.

CB-SEM was performed in order assess the structural model. The results showed an appropriate model fit (χ^2^ = 427.079, df = 173, χ^2^/df = 2.469, *p* < 0.001, NFI = 0.945, CFI = 0.966, TLI = 0.959, and RMSEA = 0.065). At the *p* < 0.05 level, all of the hypotheses were statistically supported. Particularly, the subcategories of psychological benefits, including warm glow (β = 0.522 and *p* < 0.05), self-expressive benefits (β = 0.150 and *p* < 0.05), and nature experiences (β = 0.179 and *p* < 0.05), had a positive influence on attitude. Therefore, Hypotheses 1, 2, and 3 are supported. The constructs of the TPB, including attitude (β = 0.610 and *p* < 0.05), subjective norm (β = 0.187 and *p* < 0.05), and perceived behavioral control (β = 0.261 and *p* < 0.05) positively influenced behavioral intentions, which supports Hypotheses 4, 5, and 6 (Figure 2).

### 4.4. Moderating Role of Personal Norm

A multiple-group analysis was administered in order to test the moderating role of personal norm by comparing the chi-square difference of the constrained and unconstrained models based on the degrees of freedom differences. The original sample of this study (*n* = 305) was divided into two subgroups (a high personal norm group and a low personal norm group) based on a median split of the moderator score. First, it was found that personal norm plays an important moderating role in the relationship between warm glow and attitude (Δχ^2^ = 4.350 > χ^2^ = 0.5(1) = 3.84, and df = 1). Furthermore, the current study conducted an analysis of critical ratios for differences using the AMOS program. The results of pairwise parameter comparisons indicated that coefficient comparisons from Z-score are statistically significant (*p* < 0.05), which supports that the effect of warm glow on attitude was significantly different according to the level of personal norm. Thus, Hypothesis 7a is supported. The path coefficient of the low personal norm group (β = 0.344 and *p* < 0.05) was lower than the path coefficient of the high personal norm group (β = 0.539 and *p* < 0.05). On the other hand, Hypothesis 7b (Δχ^2^ = 3.602 < χ^2^ = 0.5(1) = 3.84, and df = 1) and H7c (Δχ^2^ = 3.392 < χ^2^ = 0.5(1) = 3.84, and df = 1) are not supported (Table 4).

## 5. Discussion and Implications

### 5.1. Theoretical Implications

First, the TPB has been studied in a variety of contexts, but this study contributed to the research by applying it to the context of an eco-friendly TV home shopping broadcast for the first time. The results of this study are significant, as very few studies have researched home shopping, and even fewer studies have focused on eco-friendly home shopping. The results indicated that warm glow was positively associated with attitude (Hypothesis 1). This indicates that when consumers feel good that the home shopping broadcast is helping the environment, they are more likely to have a positive attitude toward the broadcast. Prior studies examining psychological benefits have also found warm glow to have an impact on variables such as attitude and image (e.g., [14,58,68]). For example, Boobalan et al. [42] discovered that warm glow had a significantly positive influence on attitudes toward organic consumerism in both the Indian and US markets. Therefore, this study contributed to the previous literature and filled the theoretical gap in the eco-friendly home shopping industry by observing the impact of warm glow on attitude.

Second, based on the data analysis, self-expressive benefits had a positive influence on attitude (Hypothesis 2). This means that when consumers are able to express their concerns for the environment when purchasing from an eco-friendly home shopping broadcast, they are likely to develop a positive attitude toward the broadcast. Previous studies have researched the importance of self-expressive benefits in other green industries (e.g., [41,45,69,70]). For example, Hwang and Kim [46] found that self-expressive benefits had a significantly positive influence on attitude in the edible insect restaurant industry. Although prior studies have examined the significance of self-expressive benefits in other industries or domains, this is the first study to assess an eco-friendly TV home shopping broadcast.

Third, nature experiences were revealed to have a significant effect on attitude (Hypothesis 3). The results can be interpreted as when consumers feel close to nature when purchasing products/services from the eco-friendly TV home shopping broadcast, they would develop a positive attitude toward the broadcast. This is consistent with prior studies such as Hartmann and Apaolaza-Ibanez [13] and Kaplan [71], who found that nature experiences had an influence on whether consumers developed good feelings toward a brand. Unlike prior studies, this study empirically found the significance of nature experiences on attitude in the context of eco-friendly TV home shopping.

Fourth, all of the variables of the TPB, including attitude, subjective norm, and perceived behavioral control, were found to have a significant effect on behavioral intentions. These results were consistent with previous studies (e.g., [49,50,72]), which found that the variables of the TPB impacted behavioral intentions. These results are meaningful because the impact of attitude, subjective norm, and perceived behavioral control on behavioral intentions in the eco-friendly home shopping market was observed for the first time.

Lastly, this study contributed theoretically because the moderating role of personal norm was observed in the eco-friendly TV home shopping industry. The results found that personal norm had a moderating effect on the relationship between warm glow and attitude. That is, when consumers have higher levels of personal norm, then the effect of warm glow was higher on attitude. On the other hand, when consumers have lower levels of personal norm, the effect of warm glow on attitude will be lower as well. Very few studies such as Schultz et al. [59] have researched personal norm as a moderating variable, which makes this result significant.

### 5.2. Managerial Implications

First, warm glow had a significant impact on attitude. It is necessary for TV home shopping managers to promote sales of eco-friendly products and highlight specific ways the broadcasting company contributes to the protection of the environment. Creating a separate category for sustainable or eco-friendly products on the home shopping broadcast’s website will make it easier to find sustainable products. Moreover, the TV broadcasting company should continually update the sustainable measures that they are implementing on their website, whether it be the installation of zero-emission generators or the installation of LED lighting. Eco-friendly TV home shopping broadcast companies should deliver their products, including HMR products, in sustainable packaging by using paper products, biodegradable ice packs, and air sleeves. Some local governments in the United States have even set standards requiring companies to use the least amount of recycled matter and eco-friendly materials when packaging products [73]. Home shopping brands should follow suit. These strategies would help consumers become aware of how the home shopping company is contributing to protecting the Earth and, in turn, feel good about purchasing these products or services.

Second, self-expressive benefits had a significant relationship with attitude, indicating that home shopping broadcast companies should offer products and services that are eco-friendly in order to help consumers express their concerns regarding the environment. Companies realize the importance of sustainable measures, and some have even created their own recycling programs. Brands, such as Madewell and The North Face, have recycling programs that allow consumers to drop off unwanted shoes or clothing in exchange for a voucher to the store [74]. The Nespresso coffee brand has an internal sustainability standard, AAA, that ensures the company provides sustainably sourced coffee to its consumers and even offers a capsule recycling program for consumers to participate in [75] Kiehl’s is a cosmetics brand that allows consumers to bring in empty product containers in exchange for a stamp, and when 10 stamps are collected, the company gives a travel-size product in return [74]. Eco-friendly home shopping broadcast companies will not only benefit from offering eco-friendly products and services, but offering recycling programs for consumers to participate in would help consumers feel they are proactively helping the environment.

Third, nature experiences positively influenced attitude, meaning that green advertising is also important in the context of eco-friendly TV home shopping broadcasting. Hartmann et al. [76] observed that respondents exposed to advertisements with nice nature images had similar emotional ratings to respondents exposed to real nature. Schmuck et al. [77] also found that advertisements with nature images had higher brand attitudes and purchase intentions than regular advertisements in a fictional cellphone brand. Therefore, eco-friendly TV home shopping broadcasting companies should also use nature images, such as beautiful lakes or mountains, when advertising their products in order to create a greater sense of nature experiences for the consumers. By doing so, consumers would have a favorable attitude toward the broadcasting.

Finally, personal norm was found to have a moderating role in the relationship between warm glow and attitude. This means that consumers with high levels of personal norms feel a moral obligation intrinsically to engage in eco-friendly behavior [78]. When consumers have high levels of personal norm, they will feel the effects of warm glow to a higher degree and, in turn, their attitude toward the eco-friendly TV home shopping broadcast will be greater. Managers should promote the environmentally friendly aspects of their products in advertisements to raise environmental awareness among customers. Marketing strategies should also focus on the moral obligations of customers. For example, monthly surveys could be sent to customers in order to recognize the consumers with higher levels of personal norm. Coupons for future eco-friendly product broadcasts could also be sent to consumers to remind them of their moral obligations to participate in eco-friendly practices.

## 6. Conclusions

The result of the statistical analysis revealed that all proposed hypotheses about the causal relationship within the extended TPB model were accepted. More specifically, personal norms strengthened the relationship between warm glow and attitudes.

The findings of this study provide important implications for academia and the industry. First, this paper applied the concept of psychological benefits in the context of eco-friendly TV home shopping and investigated its consequences for the first time. Second, it was also the first attempt to investigate consumers’ behavioral intentions with the extended TPB model in the context of eco-friendly TV home shopping. Third, this study deepens the framework by identifying the moderating effect of personal norm in the relationship between psychological benefits and attitudes for the first time. Moreover, the present study provides managerial implications for the eco-friendly TV home shopping industry. Managers also should highlight specific ways the company contributes to the protection of the environment in order to enhance warm glow. It is necessary to provide self-expressive benefits by offering recycling programs for consumers to participate in and present nature experiences to consumers with green advertising. Lastly, they should propose marketing strategies focused on the moral obligations of customers to enhance personal norms. These practical strategies aid in elevating attitudes toward eco-friendly TV home shopping, which, in turn, improve behavioral intentions.

Nevertheless, this study has the following limitations. First, considering this study only collected data from South Korean consumers, it is challenging to generalize the results to other countries. Future research studies in this domain should include more diversified samples from other countries. Second, the survey was conducted with respondents who had purchased HMR products in general versus a specific brand; thus, the results might be different with data collected for a specific brand. Third, the majority of the respondents were in their forties and fifties, and, thus, future studies should consider targeting other age groups to examine whether differences occur among other younger or older age groups. Fourth, personal norm is known to have a relationship with proenvironmental behavior, but very few studies have explored the moderating role of personal norm. The results of our study presented significant results, but future studies should evaluate the moderating role of personal norm in other eco-friendly industries as well. Lastly, this study employed psychological benefits as a variable. Future studies may delve even further into cultural differences and explore how the subcategories of psychological benefits, including warm glow and self-expressive benefits, may differ in individualistic and collectivistic countries.

## Figures and Tables

**Table 1:** Profile of survey respondents (*n* = 305).

Variable	*n*	Percentage
**Gender**		
Male	154	50.5
Female	151	49.5
**Age**		
20s	39	12.8
30s	75	24.6
40s	87	28.5
Over 50s	104	34.1
**Monthly house income**		
$5001 and over	48	15.7
$4001–$5000	54	17.7
$3001–$4000	52	17.0
$2001–$3000	69	22.6
Under $2000	82	26.8
**Marital** **status**		
Single	136	44.6
Married	164	53.8
Widowed/divorced	5	1.6
**Education** **level**		
Less than high school diploma	23	7.5
Associate degree	44	14.4
Bachelor’s degree	167	54.8
Graduate degree	71	23.3

**Table 2:** Confirmatory factor analysis: items and loadings.

Construct and Scale Item	Standardized Loading ^a^
**Warm glow**	
With an eco-friendly TV home shopping broadcast, I can feel good because the broadcast helps to protect the environment.	0.899
With an eco-friendly TV home shopping broadcast, I have the feeling of contributing to the well-being of humanity and nature.	0.878
With an eco-friendly TV home shopping broadcast, I can feel better because the services don’t harm the environment.	0.941
**Self-expressive benefits**	
With an eco-friendly TV home shopping broadcast, I can express my environmental concern.	0.952
With an eco-friendly TV home shopping broadcast, I can demonstrate to myself and my friends that I care about environmental conservation.	0.816
With an eco-friendly TV home shopping broadcast, I can demonstrate to myself and my friends that I care about environmental conservation.	0.852
**Nature experiences**	
An eco-friendly TV home shopping broadcast can make me feel close to nature.	0.926
An eco-friendly TV home shopping broadcast can make me think of nature, fields, forests, and mountains.	0.930
An eco-friendly TV home shopping broadcast can evoke the sensation of being in nature.	0.944
**Attitude**	
Unfavorable–favorable	0.934
Bad–good	0.962
Negative–positive	0.895
**Subjective norm**	
Most people who are important to me think I should use an eco-friendly TV home shopping broadcast.	0.973
Most people who are important to me would want me to use an eco-friendly TV home shopping broadcast.	0.960
People whose opinions I value would prefer that I use an eco-friendly TV home shopping broadcast.	0.969
**Perceived behavioral control**	
Whether or not I use an eco-friendly TV home shopping broadcast is completely up to me.	0.946
I am confident that if I want, I can use an eco-friendly TV home shopping broadcast.	0.799
I have resources, time, and opportunities to use an eco-friendly TV home shopping broadcast.	0.808
**Behavioral intentions**	
Whether or not I use an eco-friendly TV home shopping broadcast is completely up to me.	0.945
I am confident that if I want, I can use an eco-friendly TV home shopping broadcast.	0.962
I have resources, time, and opportunities to use an eco-friendly TV home shopping broadcast.	0.921
**Personal norm**	
I feel an obligation to choose an eco-friendly TV home shopping broadcast.	0.919
Regardless of what other people do, because of my own values/principles I feel that I should use an eco-friendly TV home shopping broadcast.	0.919
I feel it is important that consumers use an eco-friendly TV home shopping broadcast.	0.769
Goodness-of-fit statistics: χ^2^ = 610.629, df = 224, χ^2^/df = 2.726, *p* < 0.001, NFI = 0.932, CFI = 0.955, TLI = 0.945, and RMSEA = 0.075	

Notes. ^a^ All factor loadings are significant at *p* < 0.001. NFI, normed fit index; CFI, comparative fit index; TLI, Tucker–Lewis index; RMSEA, root mean square error of approximation.

**Table 3:** Descriptive statistics and associated measures.

	No. of Items	Mean (Std Dev.)	AVE	(1)	(2)	(3)	(4)	(5)	(6)	(7)	(8)
(1) Warm glow	3	5.24 (1.37)	0.822	**0.932** ^a^	0.705 ^b^	0.724	0.743	0.292	0.502	0.738	0.697
(2) Self-expressive benefits	3	4.29 (1.33)	0.766	0.497 ^c^	**0.907**	0.772	0.652	0.365	0.387	0.617	0.615
(3) Nature experiences	3	4.46 (1.60)	0.871	0.524	0.596	**0.953**	0.662	0.317	0.457	0.675	0.714
(4) Attitude	3	5.39 (1.45)	0.866	0.552	0.425	0.438	**0.951**	0.234	0.528	0.772	0.688
(5) Subjective norm	3	3.83 (1.60)	0.936	0.085	0.133	0.100	0.055	**0.978**	0.110	0.355	0.485
(6) Perceived behavioral control	3	5.19 (1.37)	0.729	0.252	0.150	0.209	0.279	0.012	**0.889**	0.584	0.467
(7) Behavioral intentions	3	4.91 (1.39)	0.889	0.545	0.381	0.456	0.596	0.126	**0.341**	**0.960**	**0.837**
(8) Personal norm	3	4.45 (1.38)	0.760	0.486	0.378	0.510	0.473	0.235	0.218	**0.701**	**0.904**

Notes. AVE, average variance extracted; ^a^ composite reliabilities are along the diagonal; ^b^ correlations are above the diagonal; ^c^ squared correlations are below the diagonal.

**Table 4:** Moderating role of personal norm.

Path	Unconstrained Model				Constrained Model	Tests of Moderator	
	Low Personal Norm		High Personal Norm				
	*β*	*t*-Value	*β*	*t*-Value	Δχ^2^ (346) = 652.843	χ^2^ Difference	Hypotheses
H7a WG → A	0.344	2.843 *	0.539	6.483 *	Δχ^2^ (347) = 657.193	Δχ^2^(1) = 4.350	Supported
H7b SEB → A	0.082	0.890	0.224	1.917 *	Δχ^2^ (347) = 656.445	Δχ^2^(1) = 3.602	Not supported
H7c NE → A	0.153	1.765	0.278	2.087 *	Δχ^2^ (347) = 656.235	Δχ^2^(1) = 3.392	Not supported

Notes. WG, warm glow; SEB, self-expressive benefits; NE, nature experiences; A, attitude; ns, not significant; * *p* < 0.05; Δχ2(1) = 3.84 and *p* < 0.05.

## Data Availability

Data sharing not applicable.
